# Surufatinib combined with photodynamic therapy induces ferroptosis to inhibit cholangiocarcinoma *in vitro* and in tumor models

**DOI:** 10.3389/fphar.2024.1288255

**Published:** 2024-04-05

**Authors:** Yun-Peng Huang, Yong-Xiang Wang, Hui Zhou, Zhong-Tao Liu, Zi-Jian Zhang, Li Xiong, Heng Zou, Yu Wen

**Affiliations:** Department of General Surgery, The Second Xiangya Hospital of Central South University, Changsha, China

**Keywords:** ferroptosis, Photodynainic therapy, Cholangicarcinoma, GPx4, ACSL4, organoids

## Abstract

The curative effect of single therapy for advanced cholangiocarcinoma (CCA) is poor, thus investigating combined treatment strategies holds promise for improving prognosis. Surufatinib (SUR) is a novel multikinase inhibitor that has been confirmed to prolong survival of patients with advanced CCA. Photodynamic therapy (PDT) can also ablate advanced CCA and relieve biliary obstruction. In this study, we explored the anti-CCA effect of SUR combined with PDT, and explored the underlying mechanism. We found that SUR could effectively inhibit the abilities of proliferation, migration and metastasis in CCA cells (HUCCT-1, RBE). The ability of SUR to inhibit CCA was also confirmed by the HUCCT-1 cell xenograft model in Balb/c nude mice and CCA patient-derived organoids. SUR combined with PDT can significantly enhance the inhibitory effect on CCA, and can be alleviated by two ferroptosis inhibitors (Ferrostatin-1, Deferoxamine). By detecting the level of reactive oxygen species, lipid peroxides, malondialdehyde and glutathione, we further confirmed that SUR combined with PDT can inhibit CCA cells by inducing ferroptosis. Glutathione peroxidase 4 (GPX4) belongs to the glutathione peroxidase family and is mainly responsible for the metabolism of intracellular hydrogen peroxide. GPX4 inhibits ferroptosis by reducing cytotoxic lipid peroxides (L-OOH) to the corresponding alcohols (L-OH). Acyl-CoA synthetase long-chain family member 4 (ACSL4) is a member of the long-chain fatty acid coenzyme a synthetase family and is mainly involved in the biosynthesis and catabolism of fatty acids. ACSL4 induces ferroptosis by promoting the accumulation of lipid peroxides. Both SUR and PDT can induce ferroptosis by promoting ACSL4 and inhibiting GPX4. The regulation effect is found to be more significant in combined treatment group. In conclusion, SUR combined with PDT exerted an anti-CCA effect by inducing ferroptosis. Combination therapy provides a new idea for the clinical treatment of CCA.

## Introduction

Cholangiocarcinoma (CCA) is a malignant tumor originating from bile duct epithelial cells (Brindley et al., 2021). It is the second most common type of tumor in the hepatobiliary system, accounting for 10%–15% of all primary liver malignancies (Sarcognato et al., 2021). In recent years, the incidence of CCA has been increasing globally (Da Fonseca et al., 2022). Radical surgical resection is the optimal treatment for CCA. However, due to the lack of typical clinical symptoms in the early stage, its frequent peripheral invasion, and its predilection for metastasis to lymph nodes, diagnosis often does not occur until the pernicious late stage when resection rate is lower than 35% (Rizvi et al., 2018a). Moreover, CCA is not sensitive to conventional radiotherapy and chemotherapy, and targeted therapy and immunotherapy have not achieved satisfactory therapeutic effect at present, with the 5-year overall survival rate less than 20% (Banales et al., 2020). Photodynamic therapy (PDT) is a process of killing tumor cells by activating photosensitizers with light and producing reactive oxygen species (ROS) (Itoo et al., 2022). In addition to directly acting on tumor cells, PDT can also shutdown tumor vasculature, enhance the possibility of immune recognition of tumor cells, and act on neovascular endothelial cells (Inoue et al., 2013; Yu et al., 2023). In addition, compared with traditional chemotherapy and radiotherapy, PDT has many advantages, including less harm to normal tissues, strong repeatability, and the ability to eliminate small lesions. Due to the existence of natural lacunae in the biliary tract, the combination of endoscopic retrograde cholangiopancreatography - or percutaneous transhepatic cholangioscopy-directed PDT with stent placement can rapidly ablate the advanced CCA tumor and relieve biliary obstruction (Li et al., 2021). PDT has been listed in the guidelines of the National Comprehensive Cancer Network (NCCN) of CCA (Benson et al., 2014). It has been found that PDT can kill cholangiocarcinoma cells by inhibiting cell cycle and promoting apoptosis (Yang et al., 2021). Simultaneously, multi-kinase inhibitors targeting Ras, Raf, vascular endothelial growth factor and fibroblast growth factor receptor can also generate ROS and have certain anti-CCA effects, improving the prognosis of metastatic/unresectable patients (Xie et al., 2016; Cousin et al., 2022). Sorafenib has been shown to inhibit proliferation and promote apoptosis of cholangiocarcinoma cells, and has a significant inhibitory effect *in vivo* (Sugiyama et al., 2011). In a study involving 103 patients with advanced intrahepatic cholangiocarcinoma who had previously failed treatment, 43 patients achieved partial remission after Futibatinib treatment targeting FGFR2, with a median response duration of 9.7 months, it has been approved by the FDA for the treatment of cholangiocarcinoma (Goyal et al., 2023; Roskoski, 2023). Surufatinib (SUR), a novel multi-kinase inhibitor, was first demonstrated to prolong survival in patients with advanced CCA in 2021 (Phase II, progression-free survival at 16 weeks, 46.33%) (Syed, 2021; Xu et al., 2021). SUR has a good systemic effect and long duration, which can be used to inhibit the proliferation of tumor cells after PDT. They have potential combined value by producing ROS together.

Ferroptosis is an independent programmed cell death process different from apoptosis and pyroptosis, which plays dynamic role in cell metabolism (Zheng and Conrad, 2020), redox homeostasis (Holze et al., 2018) and tumor progression (Stockwell et al., 2020). Ferroptosis is characterized by a redox state imbalance with abnormal accumulation of ROS-induced lipid peroxides (Zhang et al., 2022). The process of ferroptosis is regulated by glutathione synthesis, lipid peroxidation and ferrous ions (Fe^2+^) metabolism (Lan et al., 2022). Previous studies have confirmed that PDT can kill tumor cells by inducing apoptosis, pyroptosis, neoptosis, and ferroptosis (Zeng et al., 2022). An animal experiment in a study demonstrated the combined effect of PDT and ferroptosis (Liu et al., 2018). In addition to inducing cell apoptosis, reducing angiogenesis, and inhibiting tumor cell proliferation, many multi kinase inhibitors have been discovered as novel ferroptosis agonists in recent years. Sorafenib can induce oxidative stress and ferroptosis in hepatoma cells by inducing mitochondrial damage, inhibiting SLC7A11 and depleting glutathione (Li et al., 2021). Regorafenib also promotes ferroptosis by consuming GSH (Yan et al., 2021). No studies have reported that SUR can induce ferroptosis up till now so we chose to explore this novel concept.

In this study, we discovered that SUR can affect the migration and metastasis of cholangiocarcinoma cells as well as having a cytotoxic effect and inhibitory effect on colony formation. Furthermore, SUR was found to be superior to monotherapy in inhibiting CCA when combined with pyropheophorbide a -mediated PDT (pyro-PDT). We subsequently detected ROS, lipid peroxides, GSH, and MDA, which demonstrating that SUR combined with PDT induced ferroptosis in CCA cells. We found that the combined therapy increased the expression of ACSL4 and inhibited the expression of GPX4, which further confirmed that SUR combined with PDT could induce ferroptosis. *In vivo* experiments also confirmed the efficacy of the combination therapy. Therefore, we suggest that SUR can be used as a novel ferroptosis agonist to enhance the killing effect of PDT on CCA. Our results provide a prospective new approach for CCA clinical treatment combining PDT with SUR.

## Materials and methods

### Cell culture and cytotoxicity for treatments

HUCCT-1 and RBE cells were purchased from the Cell Resource Center of the Shanghai Academy of Biological Sciences and cultured in RPMI-1640 medium supplemented with 10% fetal bovine serum and 1% Penicillin/Streptomycin solution. CCA cells were cultured in a constant temperature incubator at 37°C and 5% CO_2_.

HUCCT-1 and RBE cells were plated on 96-well plates for 24 h before intervention. After washing twice with PBS, the CCA cells were incubated with 0–450 nM pyro and 0–45 µM SUR in 100 µL complete medium. We used complete medium containing SUR to incubate for 1–4 days, the SUR treatment group was incubated with complete medium containing 10% Cell Counting Kit-8 (MCE, HY-K0301, United States) at 37°C for 1 h, and the absorption wavelength of 450 nm was detected. After incubation for 8 h (the photosensitizer is pyro), the CCA cells of PDT group were irradiated by laser at 660 nm wavelength (BWT Beijing, Diode Laser System, China) until the total energy density reached 10J/cm^2^ (Irradiate for 1 min and 45 s under the spot size of the six-well plate). The conditions of PDT used in this study were consistent with the above. We used the same method to detect the cytotoxicity (4 h after irradiation). In the above experiments, three wells were used for each treatment.

### Colony formation experiment

HUCCT-1 and RBE cells were seeded on six-well plates with a density of 2000 cells per well. After 24 h, CCA cells were treated with SUR and PDT. After CCA cells were cultured in complete medium for 10 days, they were washed twice with PBS and fixed with 4% paraformaldehyde at 37°C for 15 min. We stained the colonies with crystal violet solution for 15 min, washed it twice with PBS, dried it naturally and took pictures of the colonies with a digital camera (iPhone X).

### Cell mobility and invasion assays

Cell mobility was measured using a wound healing system, and HUCCT-1 and RBE cells were seeded on six-well plates at a density of 5 × 10^5^ cells per well. After 24 h cultured in complete culture medium, the confluent monolayer cells scratched with a 200 μL pipette tip. CCA cells was washed three times with PBS and photographed the scratch position at 0, 12, 24, and 48 h.

CCA cells were seeded in uncoated and matrigel-coated Transwell chambers (Corning, 3422, United States) to detect migration and invasion of CCA cells, respectively. 1 × 10^5^ cells were mixed with 200 μL serum-free medium and added to the upper chamber. 600 μL 20% FBS medium was added to the lower chamber and incubated for 24 h–48 h. We used cotton swab and slightly wiped the cells in the chamber. CCA cells were fixed with 4% paraformaldehyde at 37°C for 15 min and stained with crystal violet solution for 15 min. After washed twice with PBS, we took pictures and counted them under a microscope.

### Analysis the level of reactive oxygen species and LPO

CCA cells were washed twice with PBS and incubated with DCFH-DA (Beyotime Biotechnology, S0033M, China) for 30 min and BODIPY™ 581/591 C11 (MCE, HY-D1301, United States) for 45 min in the dark. The SUR group was treated with SUR for 3 days before loading the probe. In the PDT group, the photosensitizer was incubated with CCA cells for 8 h before loading the probe. We washed the probe with PBS before PDT. CCA cells were collected and then resuspended in 500 μL PBS. We injected the cells into Flow cytometry (Beckman Coulter Epics Altra, Miami, FL, United States) and analyzed the ROS and LPO Levels.

### Analysis the content of glutathione and lipid peroxide

Followed the steps in two kits to test concentration of GSH and MDA: GSH and GSSG Assay Kit (Beyotime Biotechnology, S0053, China) and Lipid Peroxidation MDA Assay Kit (Beyotime Biotechnology, S0131, China).

### Western blot

After scraping the cells, the cells were collected by centrifugation at 800 × g. CCA cells were lysed using 60 μL of RIPA lysis buffer containing a protease inhibitor. After 30 min of lysis on ice, the lysate was centrifuged at 12,000 × g for 10 min to collect the supernatant. Subsequently, 5 × SDS loading buffer was added, and the mixture was denatured at 95°C for 5 min. The electrophoresis conditions used were: 140 V, 45 min. The PVDF membrane was activated with methanol, followed by transfer at 400 mA for 35 min. The PVDF membrane was blocked with skimmed milk prepared in TBST at room temperature for 90 min. After incubating the primary antibody overnight and incubating the secondary antibody at room temperature for 50 min. TBST was used for washing three times, ECL solution was used for development, and protein content was analyzed by photography. The primary antibodies were ACSL4 (Proteintech, 22401-1-AP, China) and GPX4 (Proteintech, 67763-1-Ig, China) with a dilution ratio of 1:1000.

### Immunohistochemical staining

Embed the tissue in paraffin and then sectioned it. Placed the sections in a 65-degree oven and baked for 50 min. Sequentially immerse the tissue sections in 100% xylene, 100% alcohol, 95% alcohol, 90% alcohol, and 75% alcohol. Use antigen retrieval solution for antigen retrieval (10 mM Tris base, 1 mM EDTA Solution, 0.05% Tween 20, pH 9.0). Complete the remaining steps according to the instructions of the immunohistochemistry kit (ZSGB-BIO, PV-9005, China). Tissue sections were stained with DAB chromogenic solution and hematoxylin staining solution. Sequentially immerse the tissue sections in 75% alcohol, 90% alcohol, 95% alcohol, 100% alcohol, 100% alcohol, and 100% xylene for dehydration. Finally, sealed the slides with neutral resin and coverslips. The dilution ratio of primary antibodies is 1:200.

### *In vivo* xenograft mouse study

The animal experiments in this study were approved by the Animal Ethics Committee of the Second Xiangya Hospital of Central South University. Twenty-eight female BALB/c nude mice aged 6–8 weeks were purchased from Vital River Laboratory Animal Technology Co., Ltd. 5 × 10^6^ HUCCT-1 cells were resuspended with 200 μL PBS and inoculated into the right subcutaneous tissue of nude mice. When the tumor size reached 80–100 mm^3^, nude mice were divided into control group (0.5% CMC-Na), low dose group (40 mg/kg mouse weight) and high dose group (80 mg/kg mouse weight). The combination experiment was divided into four groups: control group, SUR group alone (40 mg/kg mouse weight), PDT group alone (15 mg/kg mouse weight, light dose is 100 J/cm^2^, laser wavelength is 660 nm) and combination treatment group. SUR group was injected intraperitoneally for 12 days. The photosensitizer was injected intravenously at 0, 3, 6, and 9 days, and the tumor was irradiated 8–10 h after the photosensitizer injection. The tumor volume and weight of mice were measured every 2 days, and the mice were euthanized after treatment. The tumor volume calculation formula was: (width^2^ × length)/2.

### Organoid culture and activity detection

The process of harvesting and culturing organoids is as described in previous work, CellTiter-Glo 3D Cell Viability Assay was used for viability detection (Zheng et al., 2021). After adding CellTiter-Glo 3D Reagent, we shook it for 5 min and measured the relative luminescence units after 25 min of incubation. We collected tumor samples larger than 1 cm^3^ from CCA patients. Added 4–5 mL of preheated human tissue digestion solution per gram of tumor sample, digested at 37°C on a shaker for 2 h, washed and sieved with a 70 μm nylon filter membrane. Tissue was centrifuged at 8°C, 300 × g for 3 min, discarded the supernatant, and repeated this process 5 times. After cell counting, resuspended 1000 cells in 50 μL Matrigel and culture using organoid culture medium. Observed the growth status of the primary organoids under a microscope daily, and passaged them after 7 days. Hoechst 33342 and PI at a concentration of 5 μg/mL were used to stain the organoids and record the survival status of cells in the organoids.

### Statistical analysis

Statistical analysis and plotting were performed using SPSS©Statistics 25 and GraphPad Prism 8. All quantitative data was expressed as mean ± SD. ANOVA test and two-sided *t*-test were used to analyze experimental differences. *p*-value <0.05 was statistically significant.

## Result

### SUR exerts cytotoxic effects on cholangiocarcinoma cells and inhibits their proliferation, migration, and invasion

**FIGURE 1:**
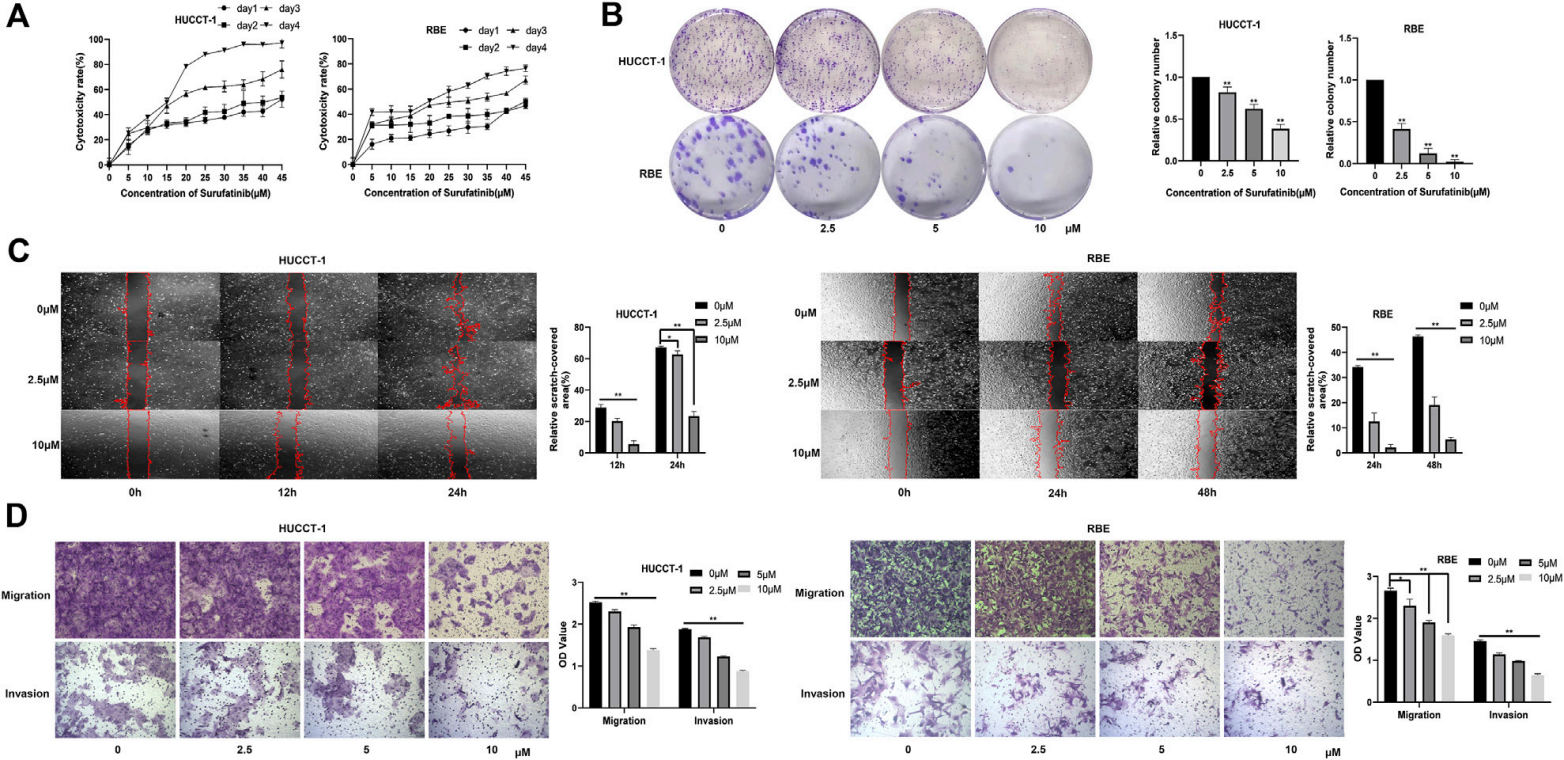
SUR exerts cytotoxic effects on cholangiocarcinoma cells and inhibits their proliferation, migration, and invasion. **(A)** HUCCT-1 and RBE cells were treated with 0–45 µM SUR for 1–4 days, and cytotoxicity was measured by CCK-8 assay. **(B)** HUCCT-1 and RBE cells were seeded in 6-well plates and treated with 0, 2.5, 5, 10 μM SUR for 10 days. **(C)** HUCCT-1 and RBE cells were treated with 0, 2.5, 10 µM SUR and cell migration was analyzed by wound healing assay at different time points (0, 12, 24, and 48 h). **(D)** HUCCT-1 and RBE cells were treated with 0, 2.5, 5, 10 µM SUR and cell migration and invasion was analyzed by transwell chamber assays. Each experiment was repeated three times, and the bar graph was shown as mean ± standard deviation. *p < 0.05 compared with 0 μM SUR, **p < 0.01 compared with 0 µM SUR.

At present, there is no basic research to explore the effect of SUR on CCA cells. Firstly, we investigated the cytotoxic effect of different time (0–4 days) and different concentration (0–45 µM) on the activity of CCA cells. The results showed that the anti-CCA effect of SUR was time-dependent and dose-dependent. From the first day to the fourth day, the half maximal inhibitory concentration (IC_50_) value of HUCCT-1 cells was found to be 53.41, 39.42, 17.44, and 11.91 µM. The IC_50_ value of RBE cells was found to be 77.65, 98.85, 24.18, and 13.89 µM (Figure 1A). According to the concentration obtained in the above experiment, three concentrations of 2.5, 5, and 10 µM were selected as experimental conditions in the subsequent experiment and treated for 3 days. The results of colony formation experiments showed that SUR could inhibit the colony formation of CCA cells with the increase of concentration (Figure 1B). Further, we explored the effect of SUR on the migration and invasion ability of CCA cell lines. The results of wound healing assay showed that SUR could significantly inhibit the migration ability of HUCCT-1 cells within 24 h (Figure 1C). Due to the weak migration ability of RBE cells, we chose 48 h as the observation point, and the results showed that the inhibition of SUR on the migration ability of RBE cells was stronger than that of HUCCT-1. Consistent with wound healing assay, transwell chamber assays demonstrated that SUR significantly reduced the migration and invasion abilities of HUCCT-1 and RBE cells (Figure 1D).

### SUR combined with PDT inhibits cholangiocarcinoma cells by inducing ferroptosis

**FIGURE 2:**
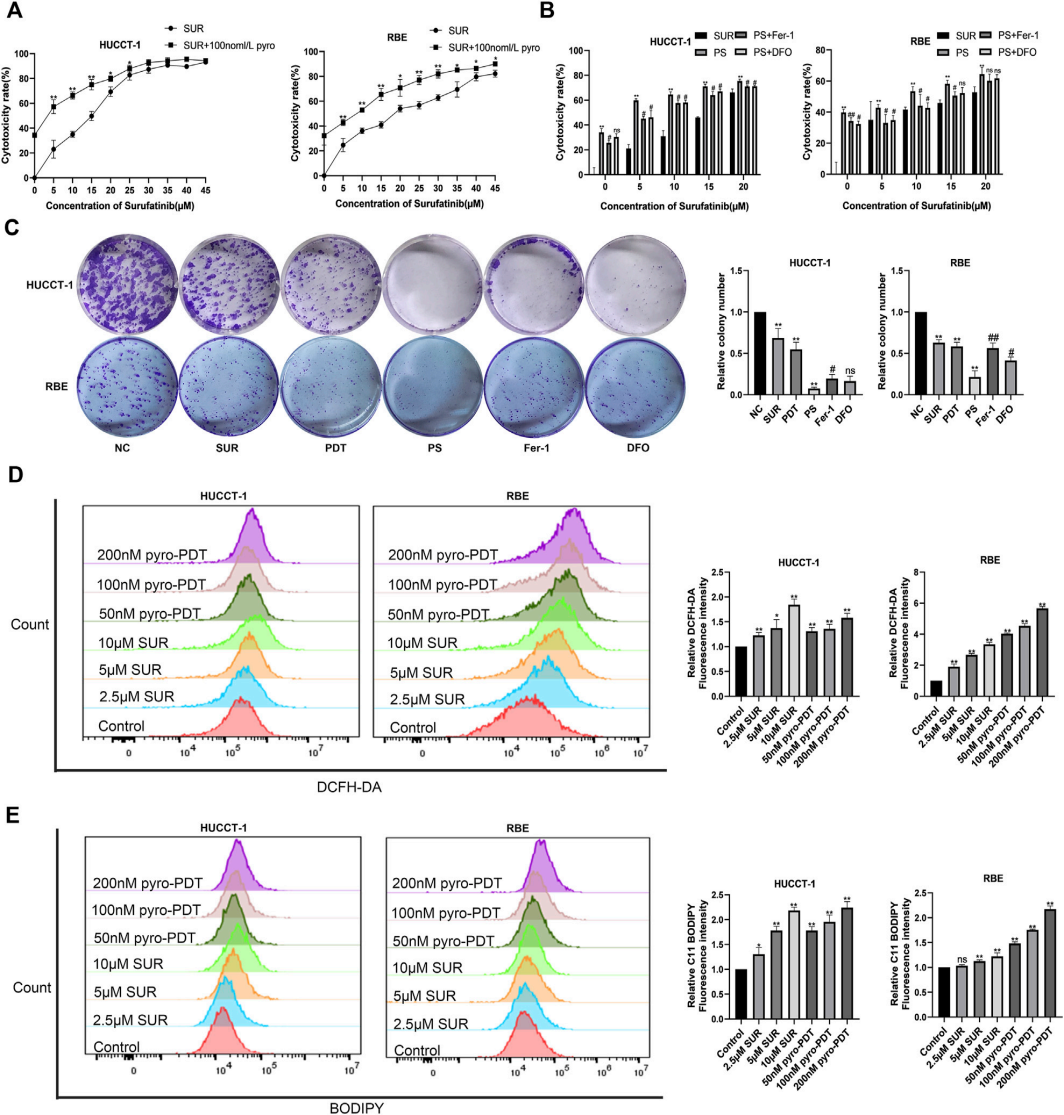
SUR combined with PDT inhibits CCA cells by inducing ROS and ferroptosis. **(A)** HUCCT-1 and RBE cells were treated with 0–45 µM SUR for 3 days, and the combination treatment group was treated with PDT (Photosensitizer concentration is 100 nM and total energy density reached 10 J/cm^2^) and different SUR concentrations. Cytotoxicity was measured by CCK-8 assay. **(B)** HUCCT-1 and RBE cells were treated with SUR (0–20 μM) and PDT. The inhibitor group was treated with Fer-1 and DFO. **(C)** HUCCT-1 and RBE cells were seeded in 6-well plates and treated with SUR (5 μM) and/or PDT (100 nM). The inhibitor group was treated with Fer-1 and DFO in addition to combination therapy. Cell proliferation was assessed by clonal formation assay. **(D)** HUCCT-1 and RBE cells were treated with SUR (0, 2.5, 5, 10 μM) and pyro- PDT (0, 50, 100, 200 nM). ROS levels were labeled using DCFH-DA probe and detected by flow cytometry. **(E)** HUCCT-1 and RBE cells were treated as above. The level of lipid peroxidation was determined with BODIPY 581/591 C11. PS means SUR combined with PDT. *p < 0.05 compared with control group, **p < 0.01 compared with control group. #*p* < 0.05 compared with PS group, ##*p* < 0.01 compared with PS group.

We verified the inhibitory effect of PDT on CCA cells and found that PDT can also exert cytotoxic effects on CCA cells in a dose-dependent manner (Supplementary Material S1). The results of colony formation experiment also confirmed the inhibition effect of PDT (Supplementary Material S2). In order to further explore the effect of SUR combined with PDT, we set up two single treatment groups and a combined treatment group respectively. The results of CCK-8 experiment showed that the cytotoxic effect of SUR combined with PDT (100 nM, 10 J/cm2) was stronger than using SUR or PDT alone (Figure 2A). In addition, two ferroptosis inhibitors, Fer-1 and DFO, can alleviate this inhibition effect (Figure 2B). The colony formation experiment also confirmed the inhibition effect of SUR and PDT alone or in combination, and Fer-1 and NAC can also reduce the inhibition effect (Figure 2C). The above results showed that SUR and PDT could inhibit CCA cells together, and this inhibitory effect could be alleviated by ferroptosis inhibitors.

The amount of ROS is an important factor for cell death caused by PDT, so we used DCFH-DA fluorescent probe to label ROS, and Annexin V-FITC assay confirmed that both PDT and SUR could produce higher concentration of ROS with the increase of dose in CCA cells (Figure 2D). Previous results using ferroptosis inhibitors suggested that SUR-induced cell death may be related to ferroptosis. Using the fluorescence probe C11-BODIPY, we observed that both SUR and PDT could increase the production of lipid peroxides in both CCA cells with a dose-dependent manner (Figure 2E).

### SUR combined with PDT promotes the expression of ACSL4 and inhibits the expression of GPX4 to induce ferroptosis

**FIGURE 3:**
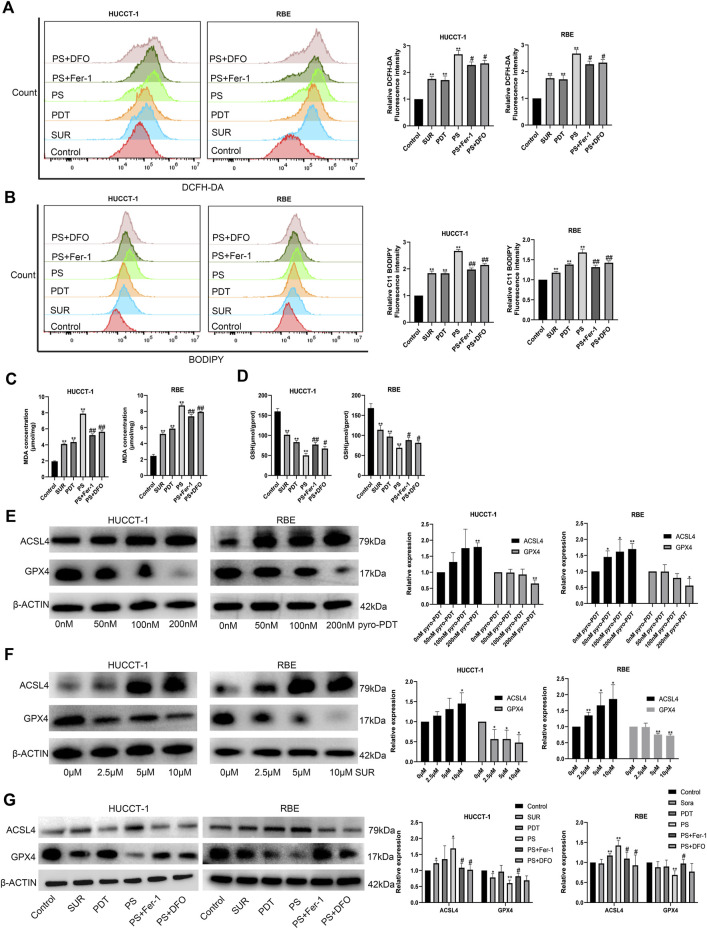
SUR combined with PDT induced ferroptosis by promoting the expression of ACSL4 and inhibiting the expression of GPX4. **(A)** The combination treatment group and the inhibitor group were treated as described above. ROS levels were tested using DCFH-DA. **(B)** Lipid peroxidation were tested using BODIPY 581/591 C11. **(C, D)** The content of MDA and GSH were measured as assay kits described. **(E, F)** After treatment with different concentrations of SUR and PDT, the expression of ACSL4 and GPX4 in HUCCT-1 and RBE cells was tested by Western Blot. **(G)** Western Blot was used to test the expression of ACSL4 and GPX4 in different groups, including control group, SUR group (5 μM), PDT group (100 nM), SUR combined PDT (PS) group and two inhibitor groups. *p < 0.05 compared with control group, **p < 0.01 compared with control group. #*p* < 0.05 compared with PS group, ##*p* < 0.01 compared with PS group.

Further analyzing the mechanism of the inhibitory effect of the combined treatment group, we found that the amount of ROS and lipid peroxides in the combined treatment group were higher than using SUR or PDT alone (Figures 3A,B). In addition, ferroptosis inhibitors can reduce the level of ROS and lipid peroxides. We detected MDA, the final product of lipid peroxidation, and found that both SUR and PDT could increase the content of MDA in the two CCA cells (Figure 3C). The MDA in the combined treatment group was higher than that in the single treatment group, and ferroptosis inhibitors could reduce this effect. Glutathione is an important antioxidant in cells and also reflects the level of ferroptosis. Compared with the single treatment group and the control group, the GSH level in the combined treatment group decreased, while ferroptosis inhibitors could slightly increase the GSH level (Figure 3D). The above results suggest that SUR combined with PDT have an anti-tumor effect by inducing ferroptosis in CCA cells.

ACSL4 and GPX4 are two classic genes regulating ferroptosis, ACSL4 is a ferroptosis promoting gene and GPX4 is a ferroptosis suppressing gene. We detected the protein changes after SUR and PDT treatment of CCA by Western Blot. The protein extraction times were: 3 days after treatment in the SUR group and 4 h after irradiation in the PDT group. The results showed that both SUR and PDT could promote the expression of ACSL4 and inhibit the expression of GPX4 (Figures 3E,F). The expression of ACSL4 in the combined treatment group was higher than that the single treatment group, while the expression of GPX4 was lower (Figure 3G). After using ferroptosis inhibitors, the effect on ACSL4 and GPX4 in the combined treatment group would be alleviated.

### SUR can inhibit the proliferation of cholangiocarcinoma *in vivo*

**FIGURE 4:**
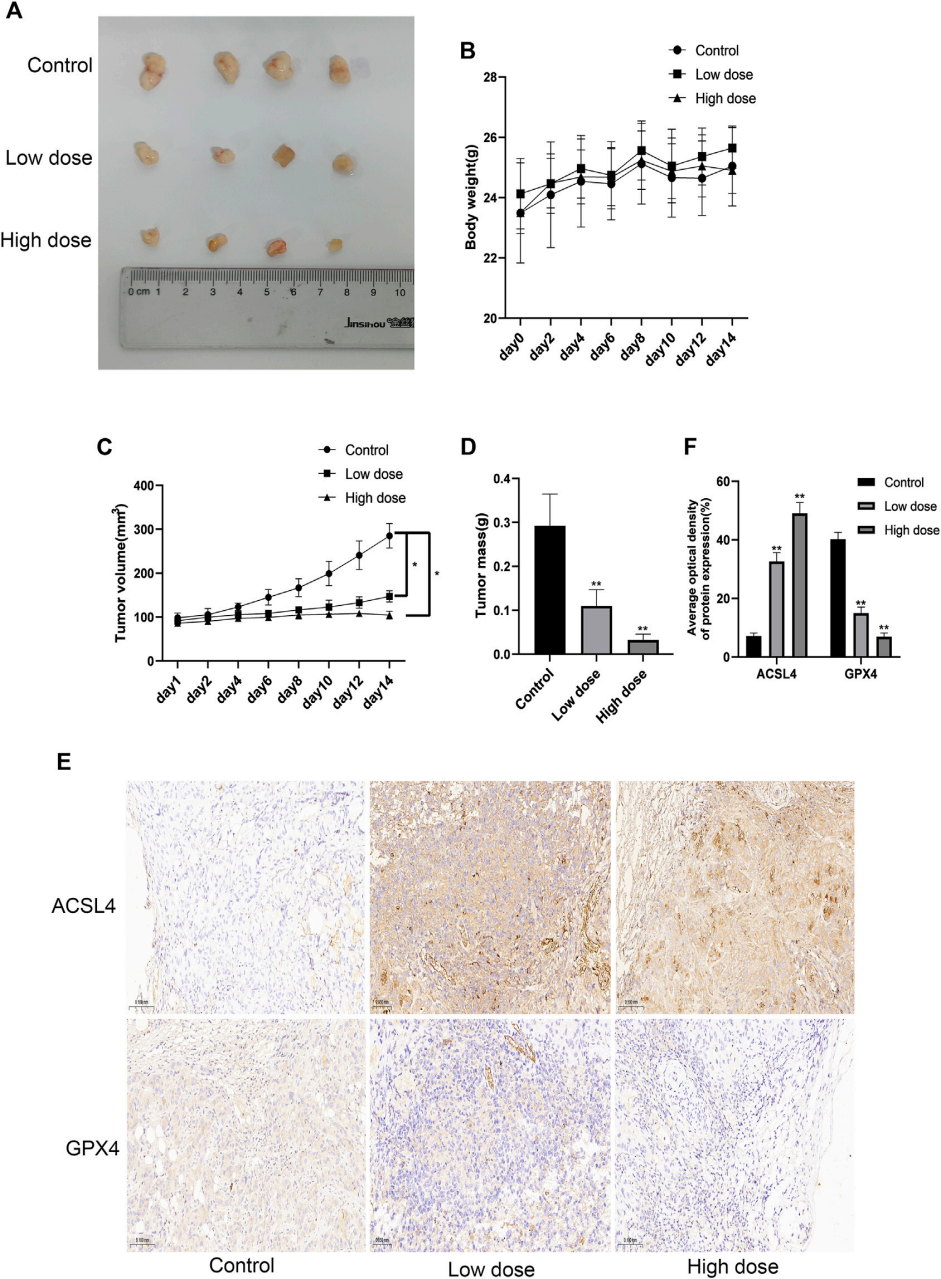
SUR inhibited the proliferation of cholangiocarcinoma *in vivo*. **(A)** When the transplanted tumor size reached 80–100 mm^3^, the mice were randomly divided into three groups (*n* = 4 in each group), and SUR (40,80 mg/kg mouse weight) was used every day. The mice were euthanized after 14 days, and the tumors were shown in the figure. **(B, C)** The weight and tumor volume of the mice were recorded every 2 days. **(D)** The weight of the tumor was measured after the mice were euthanized. **(E)** The expression of ACSL4 and GPX4 was analyzed by IHC staining. **(F)** Average optical density of ACSL4 and GPX4 expression was calculated and compared among groups.*p < 0.05 compared with control group, **p < 0.01 compared with control group.

In order to examine the safety and anti-tumor effect of SUR more comprehensively, we subcutaneously injected HUCCT-1 cells into nude mice to construct a xenotransplantation nude mice model of CCA. Nude mice were randomly divided into three groups (control group, low dose group and high dose group). After 14 days of daily intraperitoneal injection of SUR, we observed that there was no significant difference in the body weight of mice, suggesting that SUR has good biological safety (Figure 4B). The tumors of nude mice are shown in Figure 4A, the volume and weight of tumors decreased gradually with increasing SUR dose (Figures 4C,D). Through IHC detection, it was found that SUR could increase the expression of ACSL4 and decrease the expression of GPX4 (Figures 4E,F).

### SUR combined with PDT can inhibit the proliferation of cholangiocarcinoma *in vivo*

**FIGURE 5:**
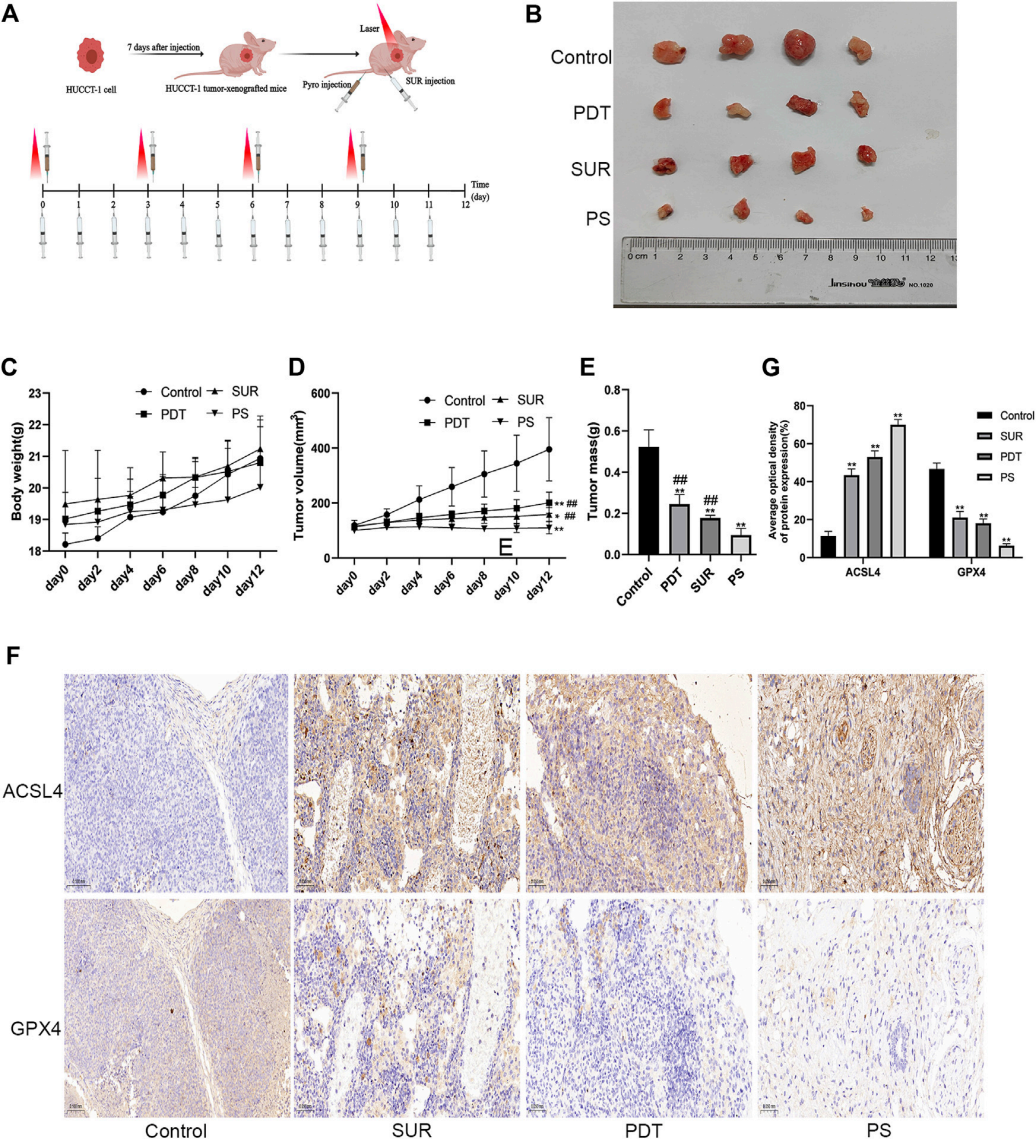
SUR combined with PDT can inhibit the proliferation of CCA *in vivo*. **(A)** Specific treatment steps of the combined treatment group. **(B)** The mice were randomly divided into four groups (*n* = 4 in each group), including control group, SUR group, PDT group and PS group. Intervention methods are as described in materials and methods. The mice were euthanized after 12 days, and the tumors were shown in the figure. **(C, D)** The weight and tumor volume of the mice were recorded every 2 days. **(E)** The weight of the tumor was measured after the mice were euthanized. **(F)** The expression of ACSL4 and GPX4 was analyzed by IHC staining. **(G)** Average optical density of ACSL4 and GPX4 expression was calculated and compared among groups.*p < 0.05 compared with control group, **p < 0.01 compared with control group. #*p* < 0.05 compared with PS group, ##*p* < 0.01 compared with PS group.

Then we further explored the inhibitory effect of SUR combined with PDT on CCA through *in vivo* experiments. The nude mice were also randomly divided into four groups (Control group, SUR group, PDT group, SUR + PDT group), and the specific treatment methods are shown in Figure 5A. After 12 days of continuous treatment, the inhibition effect of the combined treatment group was better than that of the single treatment group (Figure 5B). The volume and weight of the tumor in the combined treatment group were observed to be smaller than single treatment group, and there is no significant difference in the body weight of the mice in each group (Figures 5C–E). The expression of ACSL4 in the combined treatment group was significantly increased, and the expression of GPX4 was significantly reduced by IHC detection (Figures 5F,G). This suggested that SUR combined with PDT exerts an anti-CCA effect by inducing ferroptosis.

### SUR combined with PDT inhibits the viability of CCA organoids

**FIGURE 6:**
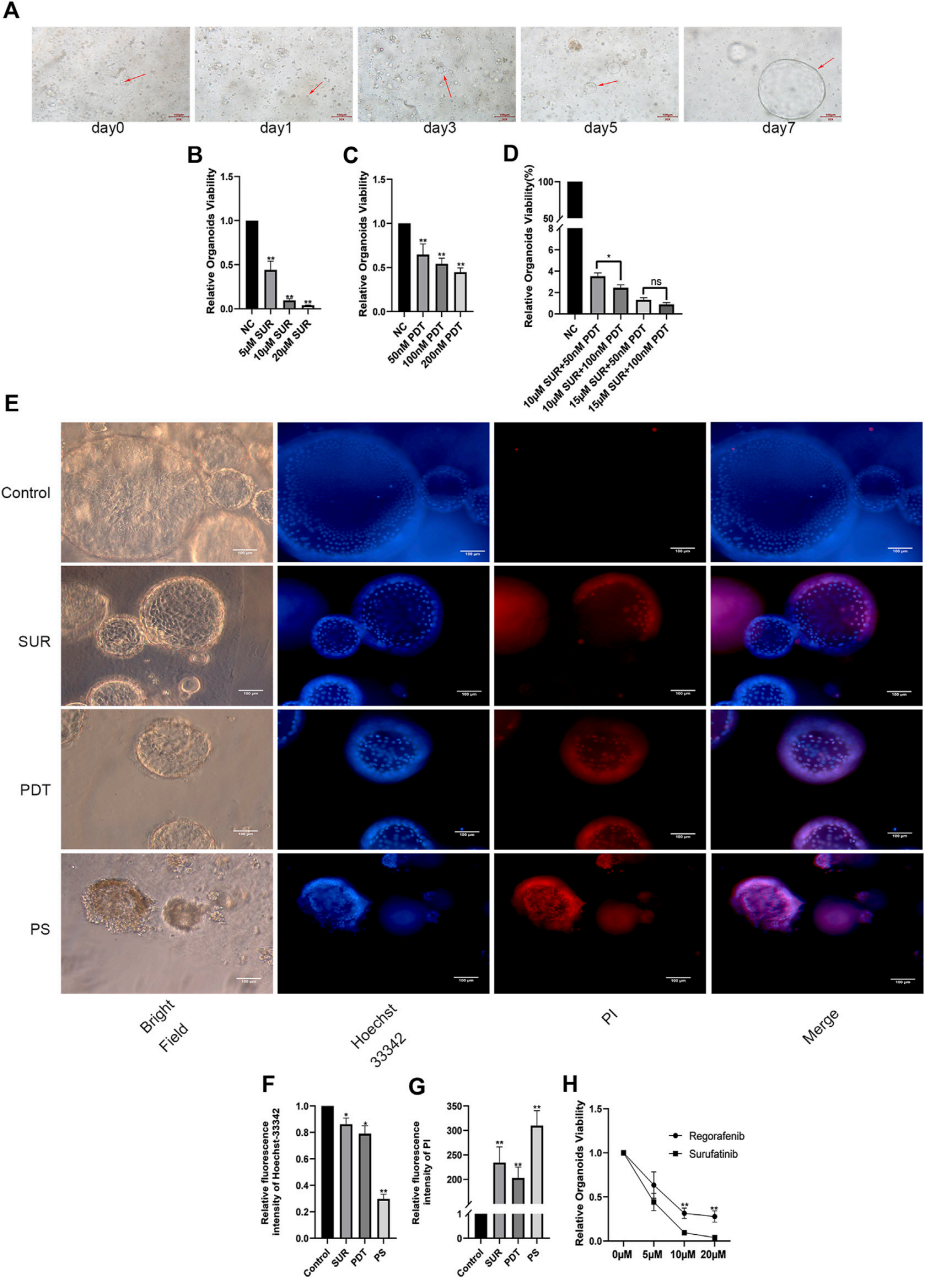
SUR combined with PDT inhibits the viability of CCA organoids. **(A)** Arrows indicate organoid growth on days 1, 3, 5, and 7. **(B–D)** Relative viability of organoids treated by different concentrations of SUR, PDT and combined treatment groups was measured by CellTiter-Glo 3D Cell Viability Assay. **(E)** Hoechst 33,342 and PI were used to label the survival status of organoids in the control group, SUR group, PDT group and the combination group. **(F,G)** Average flourescence intensity of Hoechst 33,342 and PI was calculated and compared among groups. **(H)** Compared the effects of different concentrations of Regorafenib and SUR on the vitality of CCA organoids. The viability of organoid was measured by CellTiter-Glo 3D Cell Viability Assay. *p < 0.05, **p < 0.01.

In order to verify the effect of SUR combined with PDT on patients, we constructed a CCA patient-derived organoids. Compared with the traditional subcutaneous tumor model in nude mice, patient-derived organoids maintain a three-dimensional structure and retain the characteristics of the primary tumors. Firstly, the CCA tissue was obtained from the patient. After isolation and culture, it can be observed that the organoids gradually increased from the small mass structure to the globular structure over a period of 7 days (as shown by the arrow in Figure 6A). Through CellTiter-Glo 3D Cell Viability Assay, we found that SUR and PDT had a dose-dependent inhibitory effect on the proliferation of CCA organoids, and the inhibition effect was enhanced after the combined treatment (Figures 6B–D). Then we further explored the effects of SUR (5 µM) and PDT (100 nM) on CCA organoids. We used Hochest 33342/PI method to label the organoids 4 h after irradiation, it can be observed that SUR and PDT can slightly kill CCA organoids, and the combined treatment resulted in massive cell membrane rupture and death in CCA organoids (Figures 6E–G). In addition, we compared the effects of SUR and Regorafenib on CCA organoids, and found that the inhibitory effect of SUR was better than Regorafenib (Figure 6H).

## Discussion

Over the past 40 years, the overall incidence of cholangiocarcinoma has been increasing worldwide (Aljiffry et al., 2009; Rizvi et al., 2018b). The mortality rate of cholangiocarcinoma is also increasing due to the increase of its risk factors. For example, mortality associated with intrahepatic cholangiocarcinoma has increased due to the increased incidence of chronic liver disease (Moon et al., 2020; Yan et al., 2021). Currently, long-term disease-free survival can only be achieved through surgery (liver resection or liver transplantation) (Oliverius et al., 2019). However, the 5-year overall survival rate after liver resection is 25%–40%, and approximately 50%–70% of patients have tumor recurrence (Mazzaferro et al., 2020). Therefore, it is very important to explore new clinical treatment options for CCA. Previous studies have reported that PDT mediated by ERCP or PTCS can extend overall survival time and improve the life quality of patients. Some meta-analysis also illustrated the superiority of PDT in the treatment of CCA. PDT improves survival without adverse events in patients with hilar cholangiocarcinoma (Chen et al., 2022). Mohan’s study showed that PDT has better overall survival and lower 30-day mortality than radiofrequency ablation (RFA) and/or stent-only palliative care (Mohan et al., 2022). SUR acts as a multikinase inhibitor of VEGFR 1, 2, and 3, fibroblast growth factor receptor 1 (FGFR1), and colony-stimulating factor 1 receptor (CSF1R). Some clinical trials have confirmed its efficacy in neuroendocrine tumors, thyroid tumorsand advanced biliary system tumors (Chen et al., 2020; Xu et al., 2020; Xu et al., 2021), but its underlying mechanism is still an area to be further explored. Sorafenib combined with PDT has been confirmed to have a synergistic anti-tumor effect (Sun et al., 2020). The combined treatment strategy can improve the tumor microenvironment and enhance the anti-tumor immune response. Combining with PDT is an important direction to broaden the application of sorafenib. Therefore, this study explored the inhibitory effect of SUR on cholangiocarcinoma, as well as its combined effect with PDT. In this study, the CCK-8 assay was used to confirm that SUR can significantly kill CCA cell lines (HUCCT-1, RBE), and the results of the colony formation experiment confirmed that SUR can inhibit the proliferation activity of CCA cell lines. In addition, it was found that SUR can also inhibit the invasion and migration of CCA cells by Transwell experiment. Combining SUR with PDT can further enhance the inhibitory effect on CCA cells. In addition, the results of nude mouse xenograft tumor model experiments found that SUR and PDT could inhibit tumor growth, and the inhibitory effect was more significant in the combined treatment group.

Ferroptosis is a unique mode of cell death triggered by the toxic accumulation of lipid peroxides on cell membranes, which has shown great promise in cancer therapy in recent years (Lei et al., 2022). Ferroptosis is mainly regulated by PUFA-PL synthesis and peroxidation, iron metabolism, and mitochondrial metabolism (Jiang et al., 2021). Depleting ROS and antioxidative microenvironment are the key factors for tumor cells to antagonize ferroptosis, so increasing the amount of ROS can enhance the application of ferroptosis in tumor therapy (Lin et al., 2023). ROS is also the main factor for PDT to kill tumor cells, which can lead to apoptosis, tumor tissue microvascular damage and immunogenic cell death (Ming et al., 2021). ROS is an important factor linking PDT and ferroptosis. Studies have confirmed that PDT can exert anti-tumor effects directly by inducing ferroptosis (Meng et al., 2019). Therefore, the efficacy of PDT can be enhanced by enhancing ferroptosis. Some studies have summarized the synergistic mechanism and application fields of PDT and ferroptosis in recent years (Mishchenko et al., 2021; Huang et al., 2023). PDT can produce ROS and H_2_O_2_ through type I photochemical reactions and the H_2_O_2_ can further induce ferroptosis by promoting the Fenton reaction. Multi-kinase inhibitors such as sorafenib can cause the rapid accumulation of ROS in various tumor cells, thereby inducing apoptosis and ferroptosis (Li et al., 2022). This study used DCFH-DA probe to label ROS, and found that both SUR and PDT can increase ROS levels in CCA cells through flow cytometry. The level of ROS in the combined treatment group was higher than that in the single treatment group, and the use of ferroptosis inhibitors could reduce the generation of ROS.

The balance between the oxidation and reduction states of lipids plays a critical function in the redox homeostasis, which is vital for killing tumors. According to Yang’s research, phosphorylase kinase G2 can regulate iron availability through lipoxygenase, which in turn increases the peroxidation of PUFAs and induces ferroptosis (Yang et al., 2016). Another study confirmed that polyunsaturated ether phospholipids (substrates of lipid peroxides) are inducers of ferroptosis by lipidomic profiling (Zou et al., 2020). For PDT, ^1^O_2_ produced by type II photochemical reactions can directly oxidize membrane lipids and promote LPO. Therefore, promoting LPO can enhance the efficacy of PDT. Deng prepared a plasma membrane-targeted photooxidant that can enhance LPO and promote ferroptosis after PDT (Deng et al., 2022). Increasing LPO can reverse tumor resistance and improve the chemotherapeutic effects of multikinase inhibitors. In hepatoma cells, metallothionein (MT)-1G knockdown reverses sorafenib resistance via increasing glutathione depletion and LPO-induced ferroptosis (Sun et al., 2016). In this study, C11-BODIPY probe was used to label LPO and found that SUR and PDT could promote LPO in CCA cells. The production of LPO in combined therapy group was greater than using SUR or PDT alone.

Both ferroptosis and PDT produce killing effects by inducing redox balance disorders in tumor cells. Therefore, many genes that regulate PDT may also be involved in regulating ferroptosis. Studies have found that PDT can affect the prognosis of cholangiocarcinoma patients by regulating ferroptosis-sensitive genes (Zhang et al., 2021). ACSL4 and GPX4 are two important genes that regulate ferroptosis. ACSL4 is responsible for regulating lipid metabolism, and GPX4 is involved in intracellular antioxidant regulation pathways. ACSL4 is a promoter of ferroptosis, activated by phosphorylation at Thr328, which promotes PUFA-containing lipid biosynthesis and the production of lipid peroxides, thereby inducing ferroptosis (Zhang et al., 2022). GPX4 is one of the main ferroptosis suppressor genes, which can consume GSH and reduce lipid hydroperoxide (L-OOH) to lipid alcohol (L-OH), thereby inhibiting ferroptosis (Yang et al., 2014). This study found that SUR and PDT can promote the expression of ACSL4 and inhibit the expression of GPX4, thereby promoting ferroptosis. We think that SUR can be used as a new type of ferroptosis activator, and have better anti-tumor effect when combined with PDT.

Patient-derived tumor organoids (PDO) refer to tumor cells extracted from patients’ surgical resection or biopsy samples. PDO are cultured in a medium with specific growth factors, and matrigel was used as a scaffold to construct a 3D tissue (Pasch et al., 2019). As directly obtained from tumor patients, PDO has the same histological characteristics, genetic characteristics and mutation characteristics as the parental tumors. PDO are the best alternative tools for patients to test new drugs or treatments (Jacob et al., 2020). In this study, organoids derived from cholangiocarcinoma patients were constructed to simulate the real tumor conditions of patients. The cytotoxic effect of SUR combined with PDT on cholangiocarcinoma organoids was confirmed by cell viability detection and Hoechst 3342/PI staining. The results of the combination treatment group indicate that PDT can reduce the dose of SUR required to eradicate tumors, which also means that the side effects of high SUR doses can be alleviated. Compared with the 2B drug (regorafenib) in the NCCN guidelines (Sahin et al., 2021), we found that the anti-CCA effect of SUR is better than regorafenib. This provides new evidence for the clinical research of SUR in CCA.

We can conclude the discussion by claiming that SUR can act as a new ferroptosis activator to enhance the antitumor effect of PDT on CCA. However, there are few clinical trials on SUR and PDT in the treatment of CCA, and their efficacy still needs more clinical exploration. In addition, whether combination therapy can become a new treatment strategy also needs more basic research and clinical trial verification.

## Data Availability

The raw data supporting the conclusion of this article will be made available by the authors, without undue reservation.

## Supplementary material

SUPPLEMENTARY MATERIAL PDT can kill cholangiocarcinoma cells and inhibit their proliferation. (1) HUCCT-1 and RBE cells were treated with 0-450 nM pyro and the cytotoxicity was measured by CCK-8 assay after PDT (laser at 660nm wavelength and the total energy density reached 10J/cm^2^). (2) HUCCT-1 and RBE cells were seeded in 6-well plates and treated with 0, 50, 100, 200nM pyro-mediated PDT.
