# Supplementary figures and images for: Surufatinib combined with photodynamic therapy induces ferroptosis to inhibit cholangiocarcinoma in vitro and in tumor models
Source: Front Pharmacol. 2024 Apr 5;15:1288255. doi: 10.3389/fphar.2024.1288255 (PMC11027741; doi:10.3389/fphar.2024.1288255)

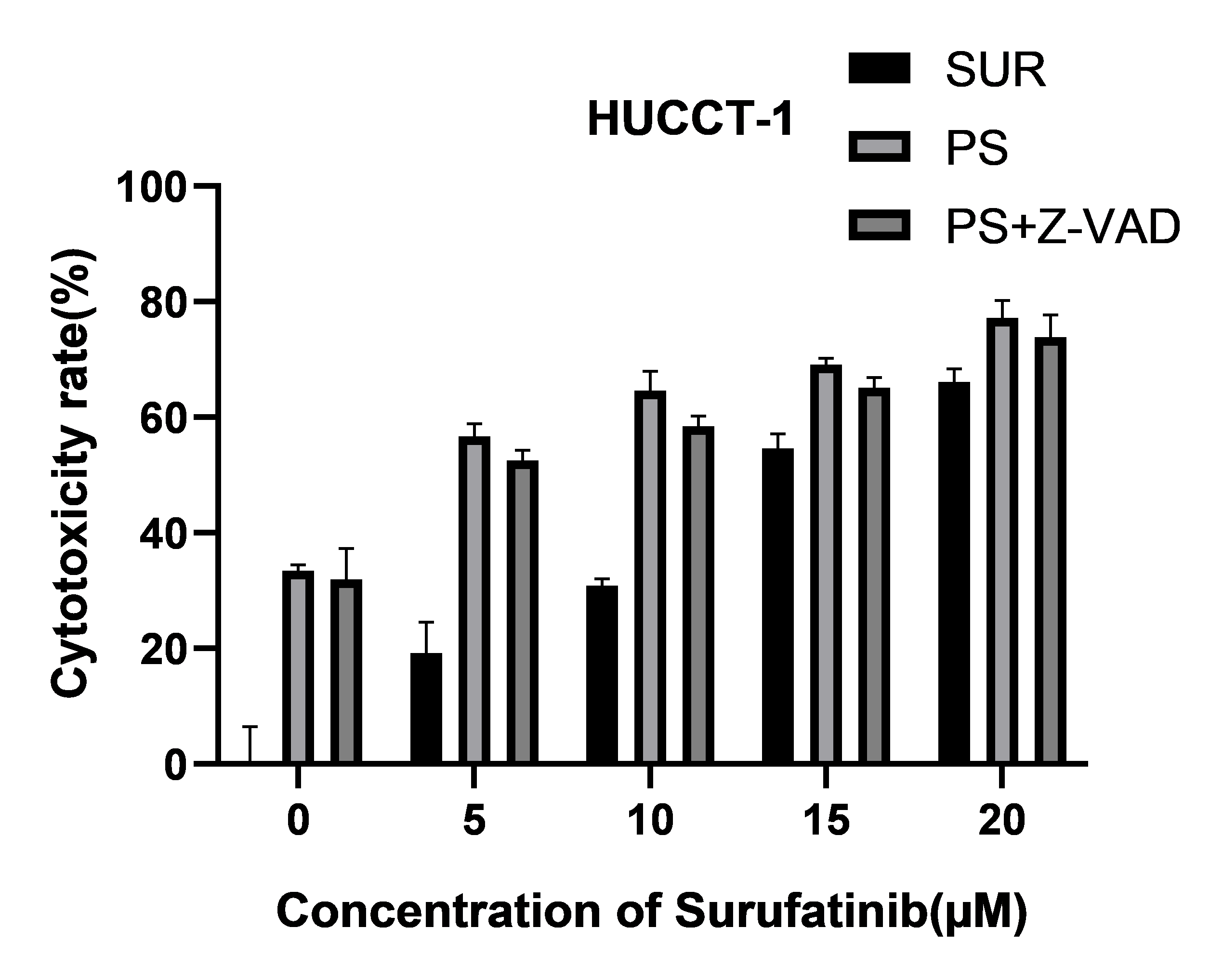

Supplement: Supplementary file 1 [file Image3.TIF]

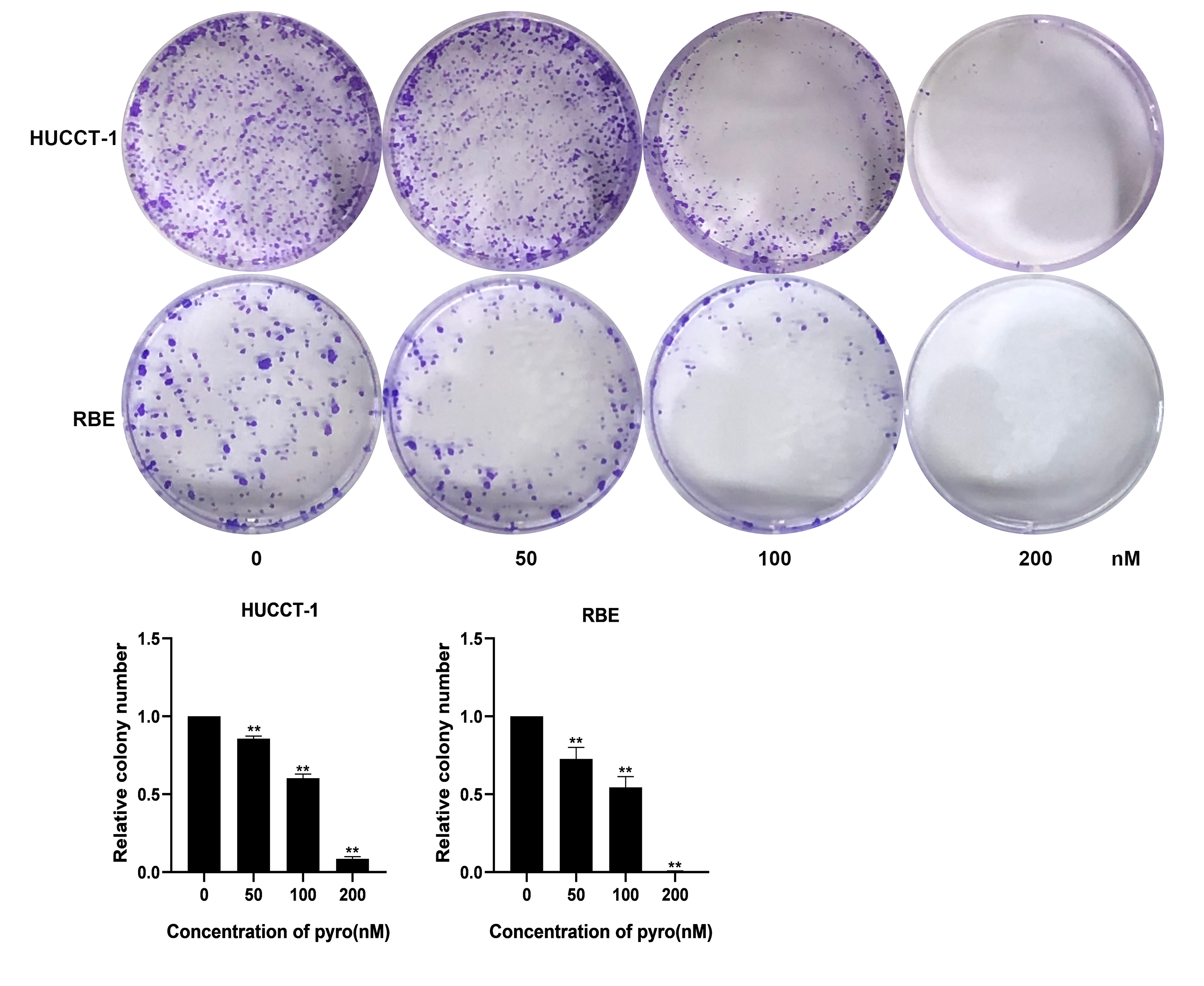

Supplement: Supplementary file 2 [file Image2.TIF]

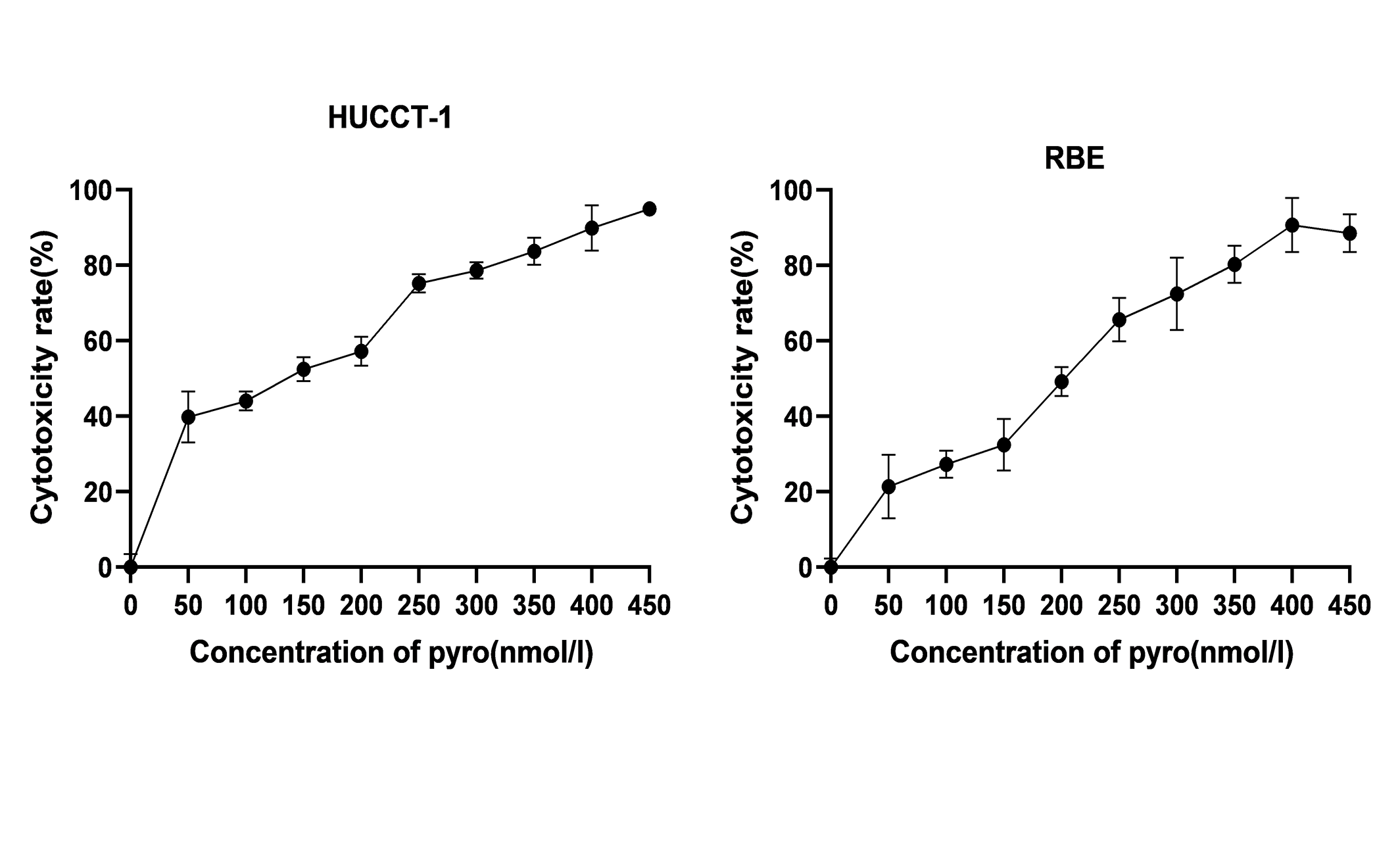

Supplement: Supplementary file 3 [file Image1.TIF]
